# *EGFR* alterations and response to anti-EGFR therapy: is it a matter of gene amplification or gene copy number gain?

**DOI:** 10.1038/bjc.2011.570

**Published:** 2011-12-20

**Authors:** R Sesboüé, F Le Pessot, F Di Fiore, T Frebourg

**Affiliations:** 1 Inserm U614, University of Rouen, Institute for Biomedical Research and Innovation, 22 Boulevard Gambetta, Rouen Cedex 76183, France; 2 Laboratory of Tumour Genetics, University Hospital, 1 rue de Germont, Rouen Cedex 76031, France; 3 Department of Pathology, University Hospital, 1 rue de Germont, Rouen Cedex 76031, France; 4 Uro-Digestive Oncology Unit, Department of Gastroenterology, University Hospital, 1 rue de Germont, Rouen Cedex 76031, France; 5 Laboratory of Molecular Genetics, University Hospital, 1 rue de Germont, Rouen Cedex 76031, France; 6 Department of Genetics, University Hospital, 1 rue de Germont, Rouen Cedex 76031, France

**Sir,**

Gene amplification, a key mechanism of oncogene activation, results from an aberrant DNA replication and leads up to several hundred of gene copies integrated either into extrachromosomal double minutes or chromosomal homogeneously staining regions. Oncogene amplification has a key role in malignant transformation, as illustrated by the canonical example of *c-myc* amplification in colorectal cancer (Masramon *et al*, 1998), and in secondary resistance to therapy, such as *MET* amplification in non-small-cell lung cancer, which leads to gefitinib resistance (Engelman *et al*, 2007). In contrast, gene copy number gain in tumours corresponds to a gene copy number >2 and may be due to numerous causes ranging from segmental chromosomal duplications to an increase of chromosome number or polyploidisation. Gene copy increase reflects the intrinsic chromosomal instability of cancerous cells and may not have any biological significance. This distinction is crucial for a critical reading of the article recently published in the journal by Ålgars *et al* (2011) entitled ‘*EGFR* gene copy number assessment from areas with highest EGFR expression predicts response to anti-EGFR therapy in colorectal cancer’. In this study, the authors first perform immunohistochemistry to assess EGFR protein expression in colorectal cancers and then determined, using silver *in situ* hybridisation, the number of *EGFR* copy and of chromosome 7 in the areas exhibiting the strongest staining. In patients without detectable *KRAS* mutation, a clinical benefit defined as partial response to anti-EGFR therapy or stable disease was observed in 23/28 (82%) of the patients with an *EGFR* copy number above 4 and in 3/16 (19%) with a lower *EGFR* copy number. Remarkably, almost the same difference was observed between patients with a high (>4.5) and low chromosome 7 number (80% *vs* 19%). Furthermore, the authors indicated that the mean value of the *EGFR*/chromosome 7 copy number ratio was 1.05 suggesting that there was no *EGFR* amplification. Although the authors indicated that *EGFR*/chromosome 7 copy number ratio was assessed, unfortunately, they did not correlate this ratio indicative of gene amplification to the anti-EGFR response. Assessment of *EGFR* gene copy number in CRC has mostly been performed using FISH, and, as highlighted by Martin *et al* (2009), the evaluation of *EGFR* FISH patterns must rely on accurate criteria in order to differentiate between a true *EGFR* amplification (*EGFR*/Chr-7 copy number ratio >2) and chromosome 7 aneusomy. If this criterion is used to define *EGFR* amplification, several studies have shown that there is a clear association between *EGFR* amplification and clinical response, with percentages of responders varying from 10 up to 89% among patients displaying *EGFR* amplification (Moroni *et al*, 2005; Frattini *et al*, 2007; Cascinu *et al*, 2008; Razis *et al*, 2008). As gene copy number increase has not the same biological significance than amplification, it is essential to distinguish these two quantitative genetic alterations, and we think that the demonstration of a real gene copy number amplification (*EGFR*/Chr-7 ratio >2) in patients’ tumour is more significant with respect to their response to monoclonal antibody-based targeted therapies.
